# The influence of ApoE4 on the clinical outcomes and pathophysiology of degenerative cervical myelopathy

**DOI:** 10.1172/jci.insight.149227

**Published:** 2021-08-09

**Authors:** Alexa Desimone, James Hong, Sydney T. Brockie, Wenru Yu, Alex M. Laliberte, Michael G. Fehlings

**Affiliations:** 1 Division of Genetics and Development, Krembil Research Institute, University Health Network, Toronto, Ontario, Canada.; 2 Institute of Medical Sciences,; 3 Division of Neurosurgery, Department of Surgery, and; 4 Temerty Faculty of Medicine, University of Toronto, Toronto, Ontario, Canada.

**Keywords:** Aging, Neuroscience, Mouse models, Surgery

## Abstract

Degenerative cervical myelopathy (DCM) is the most common cause of nontraumatic spinal cord injury in adults worldwide. Surgical decompression is generally effective in improving neurological outcomes and halting progression of myelopathic deterioration. However, a subset of patients experience suboptimal neurological outcomes. Given the emerging evidence that apolipoprotein E4 (ApoE4) allelic status influences neurodegenerative conditions, we examined whether the presence of the ApoE4 allele may account for the clinical heterogeneity of treatment outcomes in patients with DCM. Our results demonstrate that human ApoE4^+^ DCM patients have a significantly lower extent of improvement after decompression surgery. Functional analysis of our DCM mouse model in targeted-replacement mice expressing human ApoE4 revealed delayed gait recovery, forelimb grip strength, and hind limb mechanical sensitivity after decompression surgery, compared with their ApoE3 counterparts. This was accompanied by an exacerbated proinflammatory response resulting in higher concentrations of TNF-α, IL-6, CCL3, and CXCL9. At the site of injury, there was a significant decrease in gray matter area, an increase in the activation of microglia/macrophages, and increased astrogliosis after decompression surgery in the ApoE4 mice. Our study is the first to our knowledge to investigate the pathophysiological underpinnings of ApoE4 in DCM, which suggests a possible personalized medicine approach for the treatment of DCM in ApoE4 carriers.

## Introduction

Degenerative cervical myelopathy (DCM) is the most common cause of spinal cord dysfunction among adults over the age of 55 worldwide (1, 2). DCM encompasses a broad range of degenerative arthritic and congenital conditions of the cervical spine, leading to progressive cord compression and neurological dysfunction (3). DCM often presents clinically with neck and/or arm pain, weakness and numbness in the upper and lower limbs, loss of fine motor control, gait instability, and a neurogenic bladder (4). With shifting demographics and an aging population, the prevalence of DCM is expected to grow. To appropriately respond, therapeutic strategies and personalized medicine approaches to DCM treatment are critical.

Surgical decompression is currently the gold standard treatment for DCM in halting myelopathic progression, improving neurological and functional outcomes, and improving quality of life for the majority (>80%) of patients (5, 6). Although surgical decompression has shown beneficial effects, the clinical outcomes are heterogeneous. Approximately 4% of patients develop neurological complications within 30 days after decompression surgery, approximately 9.3% of patients observe a functional decline within 6 months postdecompression, and 44% of patients will experience significant residual deficits despite appropriate surgical treatment (7, 8). Numerous preoperative patient factors contribute to the level of functional improvement or impairment postdecompression, including severity of myelopathy, duration of symptoms, comorbidities, and age (9–11).

Previous studies utilizing an animal model of DCM have indicated that deterioration after decompression surgery may be caused by spinal cord ischemia/reperfusion injury (IRI) resulting in oxidative and inflammatory injury (7, 12). Perioperative IRI contributes to further damage, in part, by subsequent amplification of the inflammatory response (13–16). Activation of microglia, the resident immune cells of the central nervous system, triggers a cascade of secondary injury processes, including phagocytosis, cytokine production, antigen presentation, and neurotoxicity (17, 18). Understanding the contribution of the immune response before and after decompression surgery may be another factor to consider when determining patients’ recovery outcomes. However, key knowledge gaps remain on potential genetic risk factors that may explain the variability in postoperative recovery.

One genetic factor that has been suggested to influence outcomes of various neurological conditions is apolipoprotein E (ApoE) polymorphism (19–21). This 299–amino acid protein includes 3 common isoforms, E2 (cys112, cys158), E3 (cys112, arg158), and E4 (arg112, arg158). Each isoform differs by a single amino acid on codon 112 (rs429358) and codon 158 (rs7412), which accounts for functional differences in binding affinity to specific receptors (22). The critical role of ApoE in neurological disorders has long been studied, with significant attention on ApoE4 as a substantial risk factor for the development of Alzheimer’s disease (23, 24). It was identified that ApoE is a significant regulator of disease-associated microglia resulting in loss of their homeostatic-tolerogenic function in models of amyotrophic lateral sclerosis, multiple sclerosis, and Alzheimer’s disease (AD; 25). Numerous studies have found that the presence of ApoE4, in humans and rodent species, strongly promotes an increase in proinflammatory cytokine production in the blood, brain, and microglia during injury or disease (26–30).

In the context of DCM, it has been suggested that patients with chronic cervical spinal cord compression who develop cervical spondylotic myelopathy (CSM), a form of DCM, are more often carriers of the E4 allele compared with those with cervical canal stenosis without CSM (20). Additionally, further investigation revealed the presence of ApoE4 significantly predicts nonimprovement after spinal cord decompression surgery or significantly lower levels of improvement relative to noncarriers (21). Thus, the E4 allele may increase the risk of DCM development, as well as hinder postoperative recovery. However, the mechanisms of this potential association with the ApoE4 allele and outcomes with treatment in DCM have not been examined to date.

As such, this study investigates the impact of ApoE polymorphism on DCM progression and outcomes following surgical decompression in both human patients and an established mouse model of DCM. Our data indicate that human DCM patients who have an ApoE4 allele have a lower level of neurological improvement, and even deteriorate, after surgical decompression. In our DCM mouse model, we demonstrate that ApoE4 induces an exacerbated proinflammatory response after decompression surgery and is accompanied by poor functional outcomes following decompressive surgery. Thus, with this work we establish a potential personalized medicine paradigm to treat and monitor an at-risk ApoE4^+^ DCM patient population. These results have the potential to change current practices for the management of DCM because the molecular pathways ApoE4 induces are targetable.

## Results

### Postoperative neurological outcomes in human DCM patients differ based on ApoE allele status.

Thirty-four patients with DCM (15 females, 19 males) scheduled for surgical decompression provided written consent and were enrolled in this study (Table 1). We examined initial and follow-up modified Japanese Orthopedic Association (mJOA) scores for patients who underwent surgical decompression to determine if their ApoE allele status influenced their recovery outcome. The follow-up mJOA scores were collected 12 months after surgical decompression. Of the 34 DCM patients enrolled, 14 (41.2%) were diagnosed with mild symptoms (mJOA score 15–18) at baseline, 11 (32.3%) with moderate symptoms (mJOA score 12–14), and 9 (26.5%) with severe symptoms (mJOA score 0–11) (31).

Blood samples were collected from enrolled patients and analyzed to determine their ApoE genotype. The allelic frequencies in the study population were as follows: *E2*: 15.6%, *E3*: 96.8%, and *E4*: 18.7%. The resulting distribution of genotypes were as follows: *E2/E3*: 4 patients (12.5%), *E2/E4*: 1 patient (3.1%), *E3/E3*: 21 patients (65.6%), and *E3/E4*: 6 patients (18.7%).

**Figure 1:**
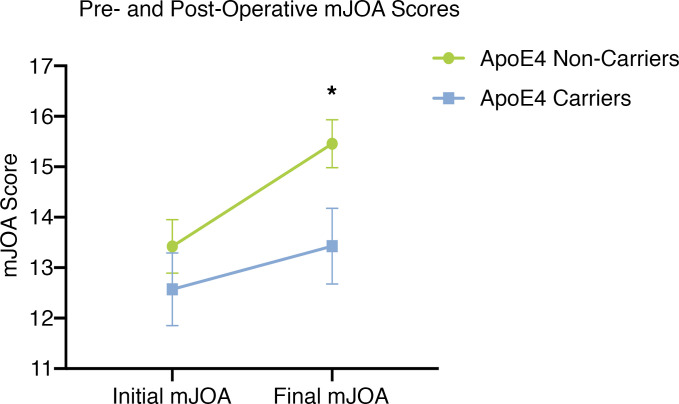
ApoE4 carriers present with a lower extent of improvement following decompression surgery when compared with ApoE4 noncarriers. ApoE4 carriers (*n* = 7) had a significantly lower mJOA score postdecompression compared with ApoE4 noncarriers (*n* = 27), *P < 0.05, Mann-Whitney *U* test. mJOA, modified Japanese Orthopedic Association.

The baseline mJOA scores of ApoE4 carriers were not significantly different when compared to noncarriers (*P* = 0.398, Mann-Whitney *U* test; Figure 1). After surgical decompression mJOA scores were significantly lower in ApoE4 carriers compared with noncarriers (*P* < 0.05, Mann-Whitney *U* test; Figure 1). Specifically, there was a greater proportion of ApoE4 noncarriers who functionally improved postdecompression compared with ApoE4 carriers (*P* = 0.013, Mann-Whitney *U* test; Table 2). The proportions of postoperative outcomes for ApoE4 noncarriers and ApoE4 carriers were as follows: improved, 17 vs. 2 (70.8% vs. 28.6%); no improvement, 7 vs. 3 (29.2% vs. 42.9%); and deteriorated, 0 vs. 2 (0% vs. 28.6%). No improvement was defined as a change of 0 or –1 points in mJOA scale (ΔmJOA = 0 or –1), and deterioration was defined as a change of at least –2 points (ΔmJOA ≤ –2).

Additionally, ApoE4 noncarriers had significantly longer duration of symptoms (*P* = 0.033, Mann-Whitney *U* test; Table 2) prior to receiving surgical decompression compared with ApoE4 carriers (ApoE4^–^ duration range: 3–36 years; ApoE4^+^ duration range: 2–7 years). However, when looking at the number of compressed segments between the ApoE4 carriers and noncarriers, there was no significant difference (*P* = 0.789, Mann-Whitney *U* test; Table 2).

### Delay in functional recovery after surgical decompression in human ApoE4–knockin mice.

**Figure 2:**
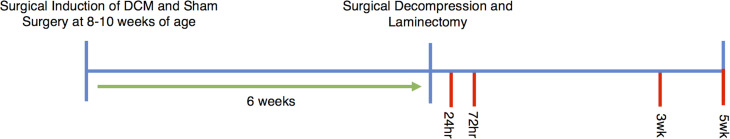
Experimental design for animal study. A schematic of crucial experimental time points. Induction of DCM or sham surgery was induced at 8–10 weeks of age in all mice and allowed for 6 weeks of gradual compression. All mice were subjected to surgical decompression with the exception of the control nondecompression group consisting of 14 total mice (4 E3-DCM; 3 E3-Sham; 4 E4-DCM; 3 E4-Sham). Multiple sacrifice time points are depicted at 24 hours, 72 hours, 3 weeks, and 5 weeks postdecompression.

B6.Cg-Apoe^em2(APOE*)Adiuj^/J (human ApoE3–knockin) and B6(SJL)-Apoe^tm1.1(APOE*4)Adiuj^/J (human ApoE4–knockin) mice were randomly and blindly assigned into DCM or sham experimental groups (E3-DCM, *n* = 20; E3-Sham, *n* = 15; E4-DCM, *n* = 21; E4-Sham, *n* = 16; Table 3) and sacrifice time points (immediately after 6 weeks of DCM [controls who received no decompression], 24 hours, 72 hours, 3 weeks, and 5 weeks postdecompression or postlaminectomy; Figure 2). Multiple sacrifice time points were utilized to capture a broad range of immune responses postdecompression. The DCM group received material implantation and surgical decompression (laminectomy and removal of the material), whereas the sham group received sham DCM surgery (no material implantation) and a laminectomy in order to control for any potential damage associated with the laminectomy that may influence postdecompression outcomes. We compared sham groups (E3-Sham and E4-Sham) with their respective age-matched DCM groups (E3-DCM and E4-DCM), as well as E3- and E4-DCM groups to identify ApoE-specific differences, pre- and postdecompression.

### Sustained gait impairments postdecompression in human ApoE4–knockin mice relative to human ApoE3–knockin mice.

**Figure 3:**
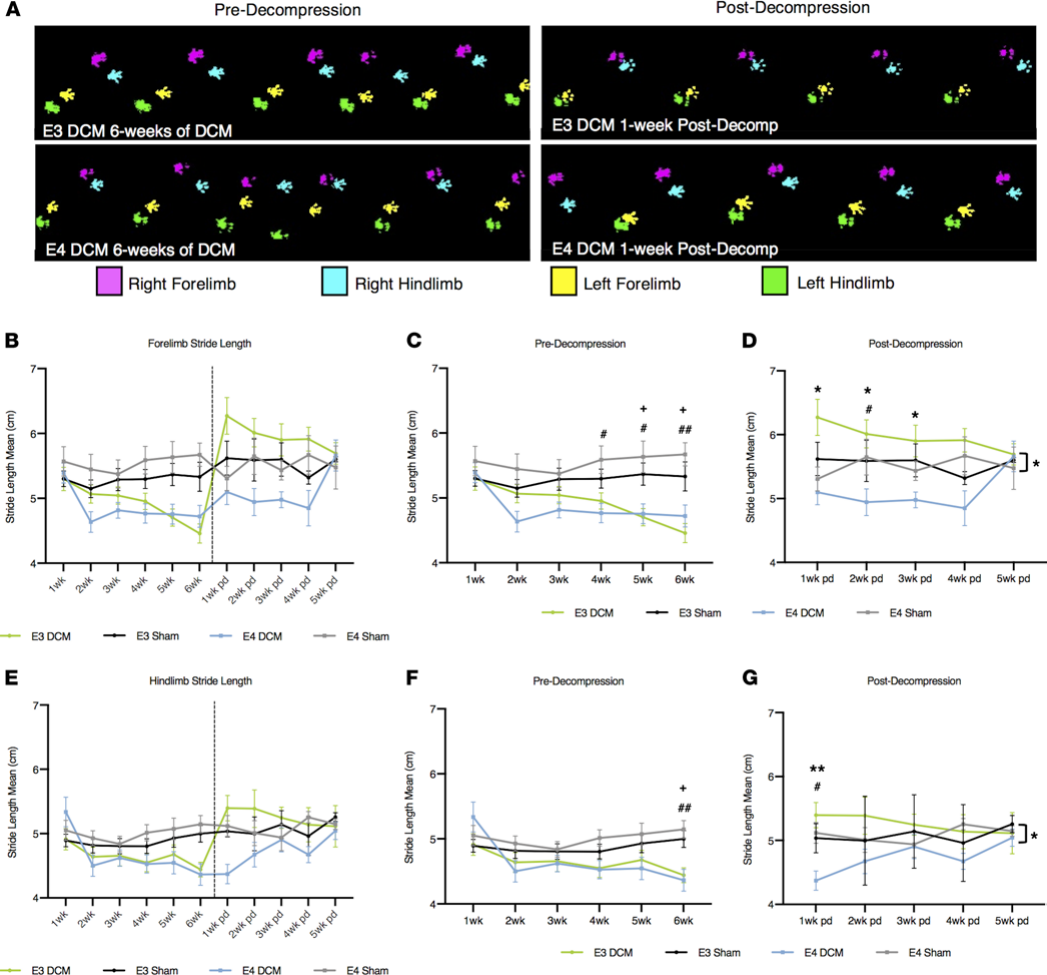
Results from gait analysis before and after decompression surgery show human ApoE4–knockin mice demonstrate a delayed recovery following decompression compared with human ApoE3–knockin mice. (**A**) Representative images of the CatWalk footprint tracking for DCM experimental groups (E3-DCM and E4-DCM) at 6 weeks postinduction of DCM and 1 week postdecompression. (**B**) Overview of forelimb stride length mean (cm) for all experimental groups pre- and postdecompression. Comparisons were made between E3-DCM and E3-Sham (indicated with + to show significance), E4-DCM and E4-Sham (indicated with # to show significance), and E3-DCM and E4-DCM (indicated with * to show significance. (**C**) Detailed forelimb stride length predecompression. ^+^*P* < 0.05, ^#^*P* < 0.05, ^##^*P* < 0.01. (**D**) Detailed forelimb stride length postdecompression. ^#^*P* < 0.05, *P < 0.05. (**E**) Overview of hind limb stride length mean (cm) for all experimental groups pre- and postdecompression. (**F**) Detailed hind limb stride length predecompression. ^+^*P* < 0.05, ^##^*P* < 0.01. (**G**) Detailed hind limb stride length postdecompression. ^#^*P* < 0.05, **P < 0.01. The overall group means of forelimb and hind limb stride length for E3-DCM and E4-DCM were significantly different postdecompression, *P < 0.05. All data are represented as mean ± SEM and were analyzed using a mixed effects model with Sidak’s post hoc correction for repeated measures.

**Figure 4:**
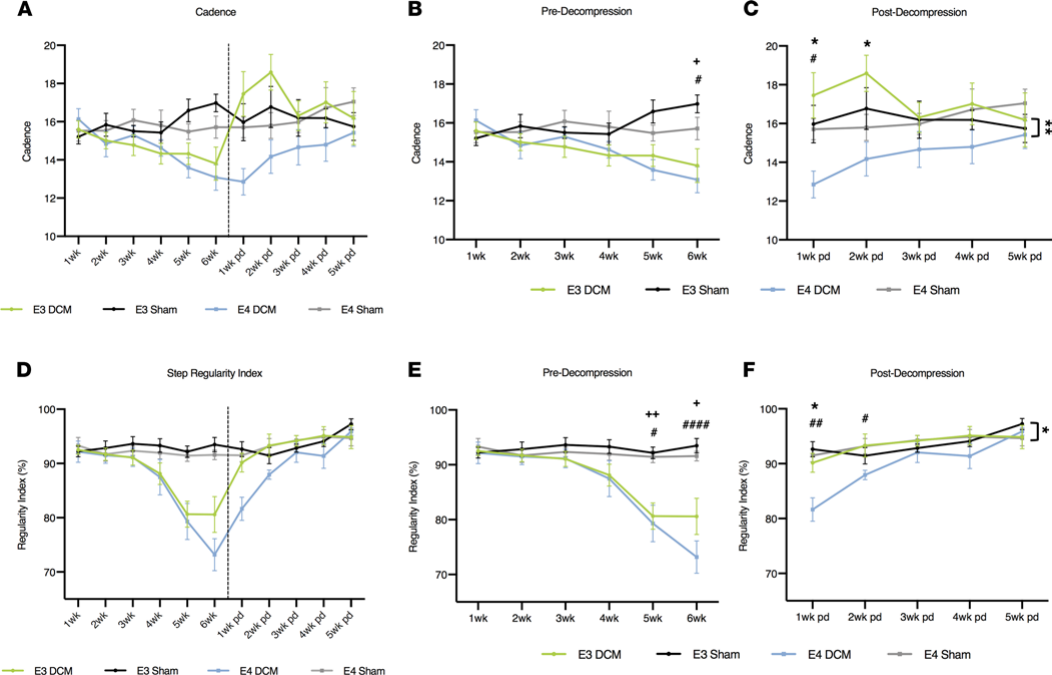
Results from gait analysis before and after decompression surgery show human ApoE4–knockin mice demonstrate a delayed recovery following decompression compared with human ApoE3–knockin mice. (**A**) Overview of cadence for all experimental groups pre- and postdecompression. (**B**) Detailed cadence predecompression. ^+^*P* < 0.05, ^#^*P* < 0.05. Comparisons were made between E3-DCM and E3-Sham (indicated with + to show significance), E4-DCM and E4-Sham (indicated with # to show significance), and E3-DCM and E4-DCM (indicated with * to show significance). (**C**) Detailed cadence postdecompression. ^#^*P* < 0.05. *P < 0.05, **P < 0.01. (**D**) Overview of step regularity index for all experimental groups pre- and postdecompression. (**E**) Detailed regularity index predecompression. ^+^*P* < 0.05, ^++^*P* < 0.01, ^#^*P* < 0.05, ^####^*P* < 0.0001. (**F**) Detailed regularity index postdecompression. ^#^*P* < 0.05, ^##^*P* < 0.01, *P < 0.05. All data are represented as mean ± SEM and were analyzed using a mixed effects model with Sidak’s post hoc correction for repeated measures. Dashed vertical line in graphs (**A** and **D**) represent the decompression time point. Wk, week; pd, postdecompression.

To investigate ApoE isoform-specific differences in gait impairments pre- and postdecompression, we performed locomotor analysis using the CatWalk (Noldus) system in all experimental groups. Gait impairment is considered one of the first symptoms of DCM; specifically, stride length and interlimb coordination are shown to be significantly affected in progressive spinal cord injury (32). Representative footprints from DCM mice at 6 weeks predecompression and 1 week postdecompression for both ApoE isoforms are shown in Figure 3A. We examined 3 gait parameters, stride length (Figure 3, B–G), cadence (Figure 4, A–C), and step regularity index (Figure 4, D–F) for differences between forelimb and hind limb, and interlimb coordination between DCM and sham groups as well as within DCM groups. Across the 3 gait parameters measured, there were no significant differences between E4-DCM and E3-DCM groups predecompression, suggesting there is a similar deterioration in both isoforms in response to DCM induction.

A shorter stride length depicts injury where the animal compensates for gait dysfunction by shortening each step. For the first 3 weeks after induction of DCM, we observed no significant difference in forelimb stride length between DCM and sham groups. However, E4-DCM and E4-Sham exhibited significant differences in forelimb stride length at 4 (*P* = 0.0247), 5 (*P* = 0.0332), and 6 weeks (*P* = 0.0036) after induction of DCM (mixed effects analysis with Sidak’s multiple comparisons; Figure 3C). E3-DCM and E3-Sham only exhibited significant differences at 5 (*P* = 0.0314) and 6 weeks (*P* = 0.0220) after induction of DCM (Figure 3C). More importantly, E3-DCM had a significantly longer forelimb stride length compared with E4-DCM at 1 (*P* = 0.0297), 2 (*P* = 0.0198), and 3 weeks (*P* = 0.0437) postdecompression, suggesting a quicker recovery (mixed effects analysis with Sidak’s multiple comparisons; Figure 3D). Additionally, at 2 weeks postdecompression, there was a significant difference between E4-DCM and E4-Sham (*P* = 0.0246). For hind limb stride length, there were significant differences between DCM and sham groups observed at 6 weeks after induction of DCM for both isoforms (E3-DCM vs. E3-Sham; *P* = 0.0193; E4-DCM vs. E4-Sham; *P* = 0.0065; mixed effects analysis with Sidak’s multiple comparisons; Figure 3F). This was followed by a significant difference between E4-DCM and E4-Sham (*P* = 0.0267), as well as E3-DCM and E4-DCM (*P* = 0.0056) 1 week postdecompression, suggesting an initial lack of recovery (mixed effects analysis with Sidak’s multiple comparisons; Figure 3G). However, it was observed that the E4-DCM group eventually recovered in both forelimb and hind limb stride length postdecompression. Specifically, there were no significant differences between the E4-DCM and E3-DCM groups in forelimb stride length at 4 and 5 weeks postdecompression and in hind limb stride length between 2 and 5 weeks postdecompression.

Interlimb coordination was investigated via cadence and step regularity index. When looking at cadence, the DCM and sham groups for both isoforms showed significant differences at 6 weeks after induction of DCM (E3-DCM vs. E3-Sham; *P* = 0.0214; E4-DCM vs. E4-Sham; *P* = 0.0345; mixed effects analysis with Sidak’s multiple comparisons; Figure 4B). Cadence measures the coordination of steps, where a higher cadence represents more coordination. At 1 week postdecompression, E4-DCM and E4-Sham depicted differences, suggesting E4-DCM delayed recovery compared with its own genetic control (*P* = 0.0378; mixed effects analysis with Sidak’s multiple comparisons). Additionally, there were significant isoform differences up until 2 weeks postdecompression between E3-DCM and E4-DCM (1 week *P* = 0.0342; 2 weeks *P* = 0.0242; Figure 4C). However, between 3 and 5 weeks postdecompression, there were no significant differences between the E4-DCM and E3-DCM groups, suggesting the E4-DCM mice eventually recovered. Step regularity index was found to be significantly lower at 5 (E3-DCM vs. E3-Sham; *P* = 0.0014; E4-DCM vs. E4-Sham; *P* = 0.0153) and 6 weeks (E3-DCM vs. E3-Sham; *P* = 0.0101; E4-DCM vs. E4-Sham; *P* < 0.0001) postinduction of DCM for both isoforms when compared with their respective sham groups (mixed effects analysis with Sidak’s multiple comparisons; Figure 4E). The regularity index measures the uniformity of the step sequence from 100% regular to 0% regular. E4-DCM and E4-Sham had significant differences up until 2 weeks postdecompression (1 week *P* = 0.0118; 2 weeks *P* = 0.0478) and isoform-specific differences at 1 week postdecompression (*P* = 0.0064; mixed effects analysis with Sidak’s multiple comparisons; Figure 4F). However, between 2 and 5 weeks postdecompression, the E4-DCM and E3-DCM groups were not significantly different, suggesting only an initial delay in recovery for the E4-DCM mice.

### Forelimb strength did not improve following decompression surgery in human ApoE4–knockin mice.

**Figure 5:**
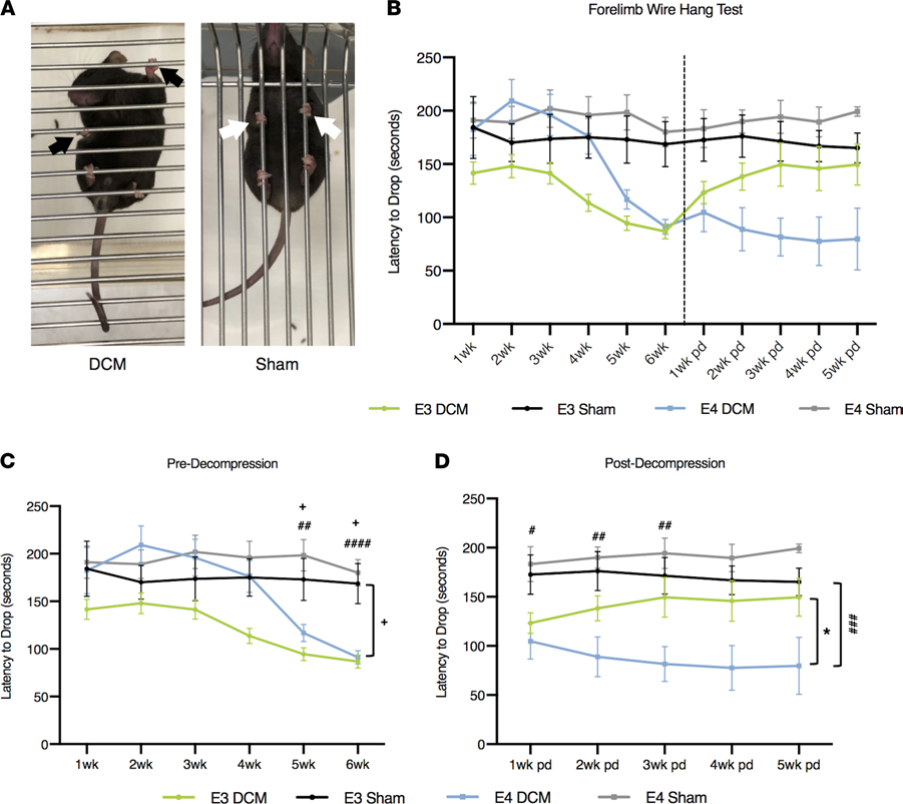
Results from forelimb grip strength before and after decompression surgery demonstrate human ApoE4–knockin mice do not improve following decompression compared with human ApoE3–knockin mice. (**A**) Representative images of DCM and sham animals prior to decompression surgery performing the wire hang test to assess forelimb grip strength. Black arrows indicate abnormal grip holding on wire grid. White arrows indicate normal grip holding on wire grid. (**B**) Overview of weekly behavioral test of forelimb grip strength via latency to drop (seconds) on the wire hang test in both transgenic mouse models of DCM and sham surgeries. Dashed vertical line represents the decompression time point. Comparisons were made between E3-DCM and E3-Sham (indicated with + to show significance), E4-DCM and E4-Sham (indicated with # to show significance), and E3-DCM and E4-DCM (indicated with * to show significance). (**C**) Detailed grip strength predecompression. E3-DCM (*n* = 35) and E4-DCM (*n* = 37) had shorter drop latencies when compared with their respective shams at 5 and 6 weeks after induction of DCM, ^+^*P* < 0.05, ^##^*P* < 0.01, ^####^*P* < 0.0001. Additionally, the overall group means for E3-DCM and E3-Sham were significantly different. (**D**) Detailed analysis of grip strength postdecompression. E3-DCM recovered to sham levels of drop latency, whereas, E4-DCM continued to be significantly more impaired when compared with E4-Sham at 1 to 3 weeks postdecompression. Overall group means were significantly different, ^#^*P* < 0.05, ^##^*P* < 0.01, ^###^*P* < 0.001. Additionally, overall group means of E3-DCM and E4-DCM differed significantly, *P < 0.05. All data are represented as mean ± SEM and analyzed via mixed effects model with Sidak’s post hoc correction for repeated measures.

Functional deficits in the upper extremities are considered another hallmark symptom of patients with DCM (4). We investigated forelimb dysfunction by measuring grip strength pre- and postdecompression using the wire hang test (Figure 5B). There were no significant differences between human ApoE3– and human ApoE4–knockin mice predecompression. Representative images of forelimb grip strategies are depicted in Figure 5A for DCM and sham animals. At 5 (E3-DCM vs. E3-Sham; *P* = 0.0254; E4-DCM vs. E4-Sham; *P* = 0.0018) and 6 weeks (E3-DCM vs. E3-Sham; *P* = 0.0134; E4-DCM vs. E4-Sham; *P* < 0.0001) after induction of DCM, E3-DCM and E4-DCM had significantly lower drop latencies compared with their respective sham groups, suggesting the DCM injury properly depicts the human symptom of upper limb weakness (mixed effects analysis with Sidak’s multiple comparisons; Figure 5C). However, this difference continued at 1 (*P* = 0.0488), 2 (*P* = 0.0067), and 3 weeks (*P* = 0.0019) postdecompression in E4-DCM compared with E4-Sham, showing an inability to recover for the E4 isoform (mixed effects analysis with Sidak’s multiple comparisons; Figure 5D). Unlike the other functional parameters measured, the E4-DCM and E3-DCM forelimb strength never converged, and the impairment seen in the E4-DCM groups was sustained up to the 5-week postdecompression time point. Additionally, there was an overall significant isoform difference between E3-DCM and E4-DCM, suggesting there is an isoform-dependent delay in limb recovery (*F*_1,14_ = 5.25; *P* = 0.0379).

### Significant dysfunction in mechanical sensitivity postdecompression in human ApoE4–knockin mice compared with human ApoE3–knockin mice.

**Figure 6:**
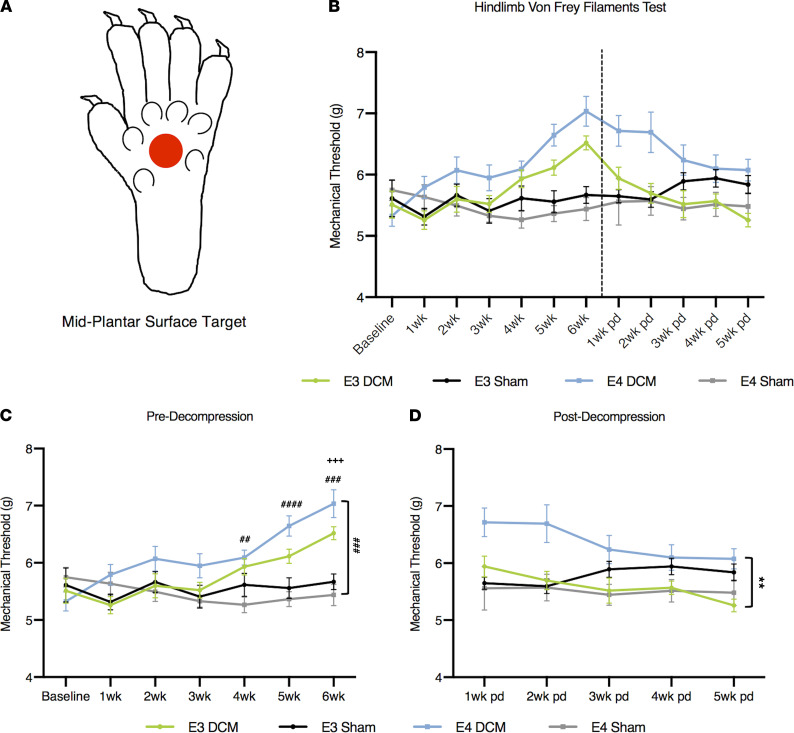
Results from hind limb mechanical sensitivity threshold before and after decompression surgery demonstrate human ApoE4–knockin mice have a delayed recovery following decompression compared with human ApoE3–knockin mice. (**A**) Representative image of probe target area (depicted by red circle) located midplantar region of the left hind paw. (**B**) Overview of hind paw mechanical sensitivity. A higher mechanical threshold depicts a lower sensitivity due to hind limb numbness caused by injury. A baseline measurement was taken prior to surgical induction of DCM or sham surgery to assess consistency between animals. Comparisons were made between E3-DCM and E3-Sham (indicated with + to show significance), E4-DCM and E4-Sham (indicated with # to show significance), and E3-DCM and E4-DCM (indicated with * to show significance). (**C**) Detailed analysis predecompression of mechanical sensitivity. E3-DCM and E4-DCM required a significantly higher force threshold (g) to respond compared with their respective shams at 4 to 6 weeks postinduction of DCM, ^+++^*P* < 0.001, ^##^*P* < 0.01, ^###^*P* < 0.001, ^####^*P* < 0.0001. Additionally, the overall group means for E4-DCM and E4-Sham were significantly different, ^###^*P* < 0.001. (**D**) Detailed analysis postdecompression of mechanical sensitivity. Overall group means for E3DCM and E4-DCM were significantly different, suggesting E3-DCM recovery, **P < 0.01. All data are represented as mean ± SEM and analyzed via mixed effects model with Sidak’s post hoc correction for repeated measures.

To further identify functional deficits in E3-DCM and E4 DCM mice, we investigated hind limb mechanical sensitivity pre- and postdecompression using an electric von Frey apparatus (Figure 6B; ref. 33). We targeted the midplantar region on the inferior surface of the hind paw with filamentous probes of increasing force (g) until they sufficiently stimulated the withdrawal of the paw (Figure 6A). At 4 to 6 weeks after induction of DCM, the E4-DCM group required a significantly greater force to produce a response compared with E4-Sham (4 weeks *P* = 0.0009, 5 weeks *P* < 0.0001, 6 weeks *P* = 0.0001; Figure 6C). However, only at 6 weeks after induction of DCM, this same significant difference was observed between E3-DCM and E3-Sham (*P* = 0.0004; mixed effects analysis with Sidak’s multiple comparisons; Figure 6C). After decompression, there were significant differences in overall genotype group means when comparing E3-DCM and E4-DCM, suggesting an overall isoform-dependent recovery in hind limb mechanical sensitivity (*F*_1,14_ = 15.26; *P* = 0.0016; mixed effects analysis with Sidak’s multiple comparisons; Figure 6D).

### A significant decrease in gray matter area in the human ApoE4–knockin mice 24 hours after decompression surgery.

**Figure 7:**
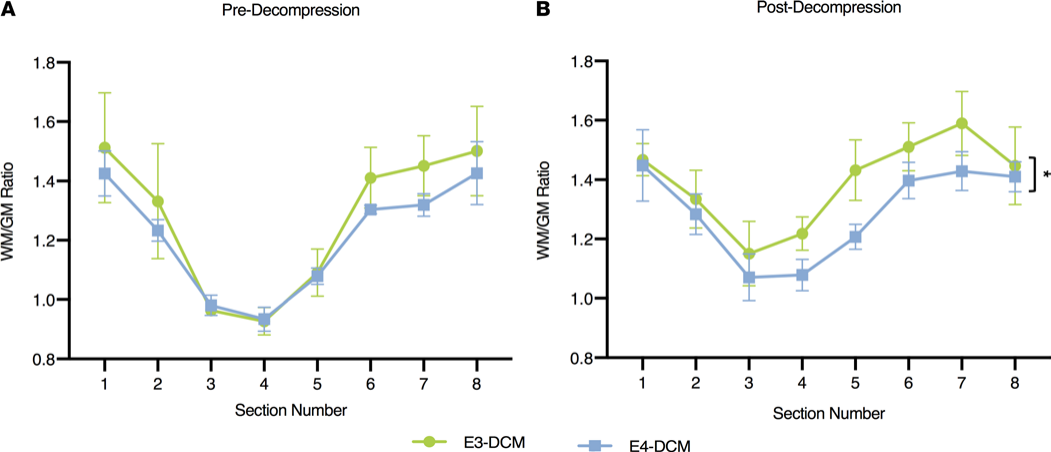
Significant decrease in gray matter area 24 hours postdecompression in human ApoE4–knockin mice when compared with their ApoE3 counterparts. (**A**) The WM/GM ratio predecompression for E3-DCM and E4-DCM mice across all 8 spinal cord sections, at a 240 μm interval around the injured epicenter. (**B**) The WM/GM ratio 24 hours postdecompression for E3-DCM and E4-DCM mice showed a significant difference between the overall group means, *P < 0.05. All data are represented as mean ± SEM and analyzed via a 2-way ANOVA, Sidak’s post hoc. WM, white matter; GM, gray matter.

To determine the extent of injury before and after decompression surgery in the human ApoE4– and ApoE3–knockin mice, we measured the gray and white matter area on sections stained with hematoxylin-eosin (HE) and Luxol Fast Blue (LFB). It was determined that before decompression, there was no significant difference between E3-DCM and E4-DCM mice (*F*_1,48_ = 1.604; *P* = 0.2144; 2-way ANOVA; Figure 7A). However, 24 hours after decompression surgery, there was a significant decrease in gray matter area for the E4-DCM mice. As such the E3-DCM had a better recovery than the E4-DCM (*F*_1,48_ = 6.034; *P* = 0.0177; 2-way ANOVA; Figure 7B).

### Exacerbated peripheral proinflammatory response 24 hours following surgical decompression in human ApoE4 knockin mice.

**Figure 8:**
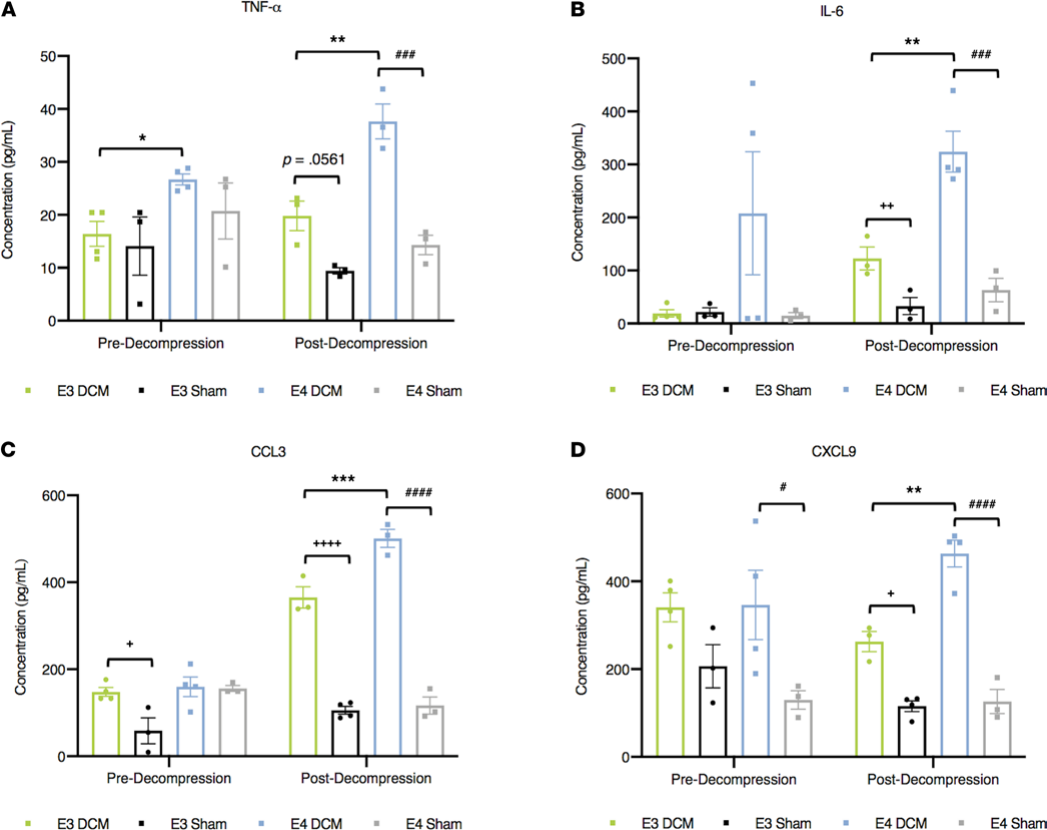
Exacerbated peripheral proinflammatory response 24 hours following surgical decompression in ApoE4-knockin mice. Using Luminex xMAP technology, concentrations of cytokines were determined. Comparisons were made between E3-DCM and E3-Sham (indicated with + to show significance), E4-DCM and E4-Sham (indicated with # to show significance), and E3-DCM and E4-DCM (indicated with * to show significance). (**A**) TNF-α concentration (pg/mL) before decompression (control) and 24 hours postdecompression for all experimental groups. Before decompression, E4-DCM had significantly higher levels of TNF-α, which continued 24 hours postdecompression, *P < 0.05, **P < 0.01; 1-way ANOVA, Sidak’s post hoc. Additionally, E4-DCM and E4-Sham had significantly different TNF-α concentrations 24 hours postdecompression, ^###^*P* < 0.001; 1-way ANOVA, Sidak’s post hoc. (**B**) IL-6 concentration (pg/mL) for E4-DCM was significantly higher compared with E3-DCM 24 hours postdecompression, with each DCM group significantly different from sham counterparts, **P < 0.01, ^++^*P* < 0.01, ^###^*P* < 0.0001; 1-way ANOVA, Sidak’s post hoc. (**C**) CCL3 concentration (pg/mL) before decompression showed E3-DCM levels significantly higher compared with E3-Sham, ^+^*P* < 0.05; 1-way ANOVA, Sidak’s post hoc. Additionally, 24 hours postdecompression E3-DCM and E4-DCM were significantly different, as well as compared with their respective sham groups, ***P < 0.001, ^++++^*P* < 0.0001, ^####^*P* < 0.0001; 1-way ANOVA, Sidak’s post hoc. (**D**) CXCL9 concentration (pg/mL) before decompression exhibited significant differences between E4-DCM and E4-Sham, ^#^*P* < 0.05; 1-way ANOVA, Sidak’s post hoc. After decompression both DCM groups were significantly different compared with their respective shams, and E4-DCM had significantly higher levels compared with E3-DCM, **P < 0.01, ^+^*P* < 0.05, ^####^*P* < 0.0001; 1-way ANOVA, Sidak’s post hoc. All data are represented as mean ± SEM.

To determine the peripheral inflammatory response before and after decompression in our DCM mouse model, serum was collected to assess cytokine levels at 2 experimental time points (predecompression and 24 hours postdecompression). Of the 31 cytokines analyzed, TNF-α (*P* = 0.0011), IL-6 (*P* = 0.0045), CCL3 (*P* = 0.0004), and CXCL9 (*P* = 0.0011) were significantly increased in the E4-DCM group compared to the E3-DCM group at 24 hours postdecompression (1-way ANOVA with Sidak’s multiple comparisons; Figure 8, A–D). Additionally, there were isoform-specific differences predecompression only in TNF-α levels, where E4-DCM had significantly higher levels compared with E3-DCM (*P* = 0.0149; 1-way ANOVA with Sidak’s multiple comparisons).

### Activation of macrophages/microglia is more prominent in human ApoE4–knockin mice 24 hours after decompression surgery.

**Figure 9:**
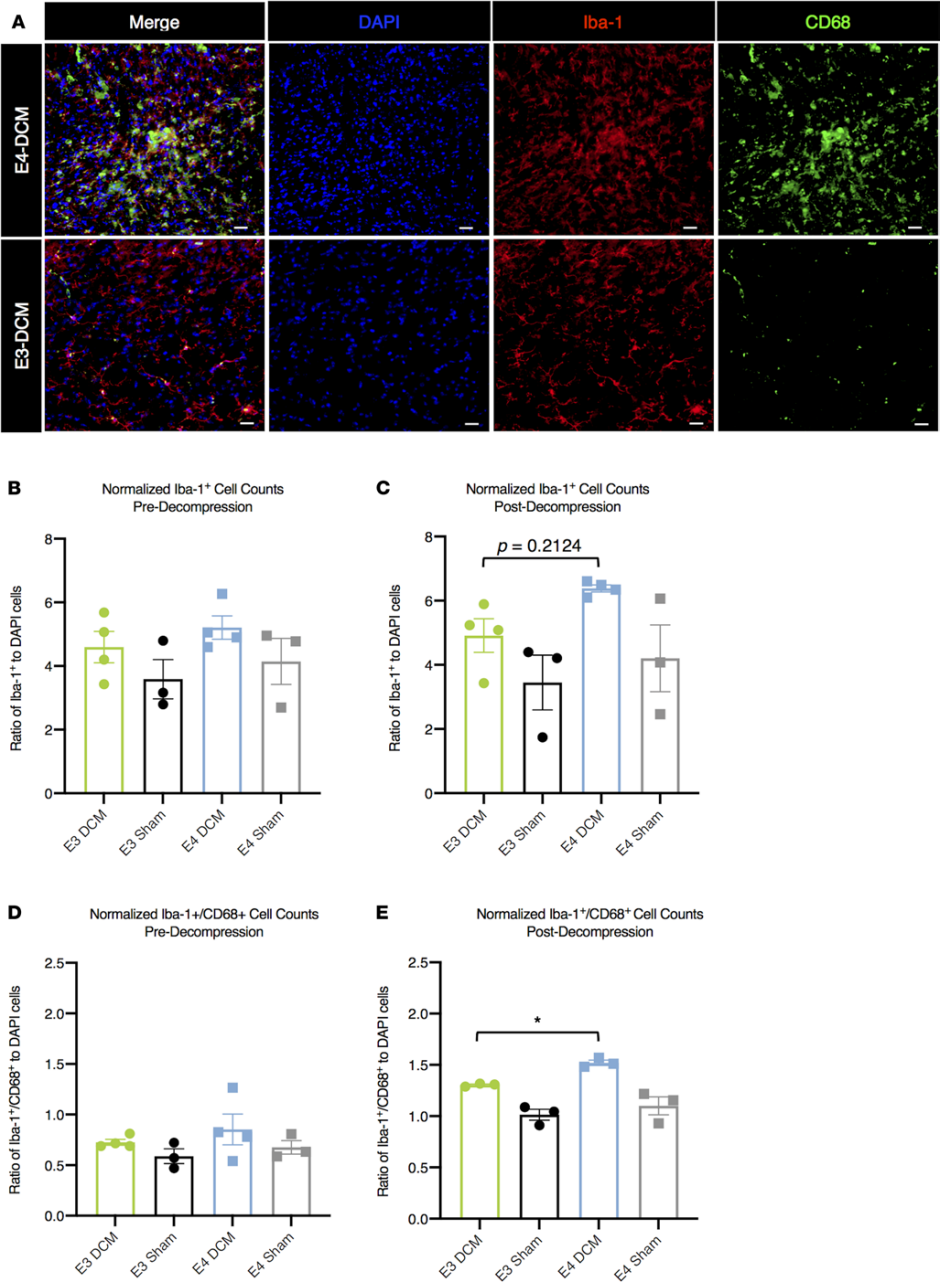
Increased activation of macrophage/microglia 24 hours after decompression surgery in human ApoE4–knockin mice. (**A**) Representative images of Iba-1^+^ (red) and CD68^+^ (green) cells with nuclear counterstain DAPI (blue) in the dorsal horns from E3-DCM and E4-DCM experimental groups 24 hours after surgical decompression. Scale bars: 20 μm. (**B**) Quantification of Iba-1^+^ cells normalized to total DAPI counts predecompression showed no significant difference across all experimental groups. (**C**) Quantification of Iba-1^+^ cells normalized to total DAPI counts postdecompression in all experimental groups. There was no significant difference in macrophage/microglial cell recruitment in E4-DCM mice compared with E3-DCM mice, as well as between the E3-Sham and E4-Sham groups. (**D**) Colocalization of Iba-1^+^CD68^+^ cells quantified and normalized to total DAPI counts predecompression showed no significant difference across all experimental groups. (**E**) Colocalization of Iba-1^+^CD68^+^ cells quantified and normalized to total DAPI counts postdecompression for all experimental groups. When Grubbs’s outlier test (α = 0.05) was performed, 2 data points were identified and subsequently removed (1 from the E3-DCM group and 1 from the E4-DCM group). Analysis determined that the E4-DCM mice had significantly more colocalization compared with E3-DCM; however, there was no significant difference between the E3-Sham and E4-Sham groups. All data are presented as mean ± SEM of 8 sections per animal (250 μm intervals between each section). These images are not the same images used for quantification; see Supplemental Figure 1 (supplemental material available online with this article; https://doi.org/10.1172/jci.insight.149227DS1) for representative images. *P < 0.05; 1-way ANOVA, Sidak’s post hoc.

To investigate whether the ApoE4 genotype influenced the recruitment and activation of macrophage/microglial cells predecompression and 24 hours after decompression surgery, we performed double-labeled immunohistochemistry on spinal cord sections around the injury epicenter with an ionized calcium-binding adapter molecule 1 (Iba-1) marker and CD68 marker for active phagocytosis (34). Cell counts for Iba-1^+^ and colocalization of Iba-1^+^CD68^+^ were normalized to DAPI cell counts and presented as a ratio. Representative images are shown from E3-DCM and E4-DCM experimental groups taken from dorsal horn regions (Figure 9A).

It was determined that predecompression there was no significant difference in the number of Iba-1^+^ cells between E3-DCM and E4-DCM mice (*P* = 0.6476; 1-way ANOVA with Sidak’s multiple comparisons; Figure 9B). Additionally, there was no significant difference postdecompression between the number of Iba-1^+^ cells in the E4-DCM experimental group compared to the E3-DCM experimental group (*P* = 0.2124; 1-way ANOVA; Figure 9C). Both before and after decompression, there was no significant difference in the number of Iba-1^+^ cells between E3-Sham and E4-Sham groups.

When investigating the colocalization of Iba-1^+^CD68^+^ cells, there were no significant differences between E3-DCM and E4-DCM groups before decompression (*P* = 0.5822; 1-way ANOVA; Figure 9D). However, postdecompression there were significant differences between E4-DCM and E3-DCM groups, such that a greater proportion of colocalization was observed in E4-DCM animals (*P* = 0.0407; 1-way ANOVA; Figure 9E). This suggests an increase in phagocytotic macrophage/microglial cells present in the E4-DCM cord when compared with E3-DCM animals. Before and after decompression, it was determined that there was no significant difference in the colocalization of Iba-1^+^CD68^+^ cells between E3-Sham and E4-Sham groups.

### Increased astrogliosis in human ApoE4–knockin mice 24 hours after decompression surgery.

**Figure 10:**
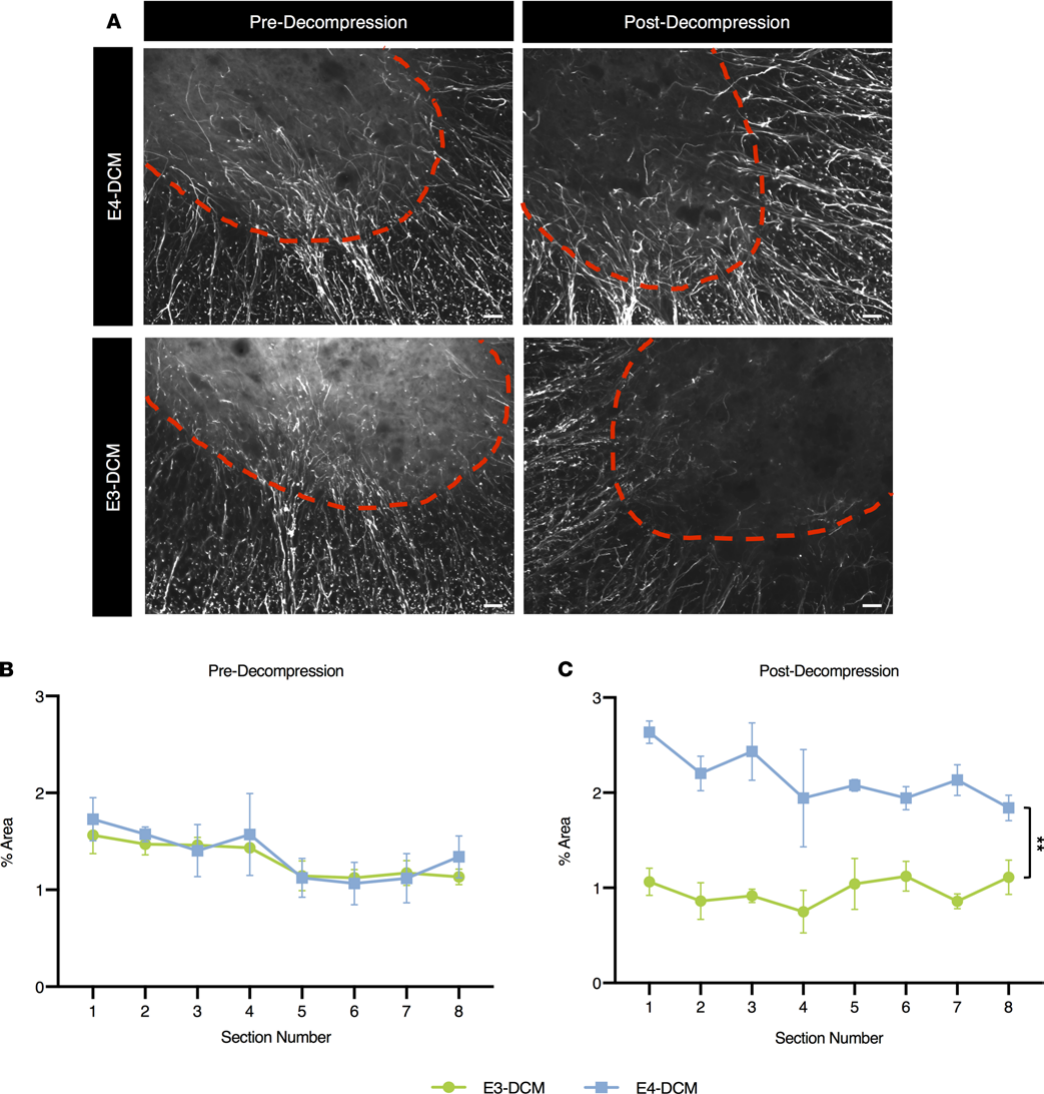
Increased astrogliosis in human ApoE4–knockin mice 24 hours after decompression surgery. Quantification of GFAP immunoreactivity in the ventral horn region of E3-DCM and E4-DCM mice was normalized to their respective shams. (**A**) Representative images of the ventral horns for E3-DCM and E4-DCM mice where GFAP immunoreactivity was quantified over an area of constant size. The red dashed line delineates the GM. (**B**) GFAP immunoreactivity predecompression in E3-DCM and E4-DCM mice showed no significant difference across the 8 cervical spinal cord sections at a 240 μm interval around the injured epicenter. (**C**) GFAP immunoreactivity in E3-DCM and E4-DCM mice was significantly different postdecompression across the 8 cervical spinal cord sections at a 240 μm interval around the injured epicenter; **P < 0.01. All data are represented as mean ± SEM and analyzed via mixed effects model with Sidak’s post hoc correction for repeated measures. These images are not the same images used for quantification; see Supplemental Figure 2 for representative images. Scale bars: 25 μm.

Together with microglia/macrophage cells, astrocytes play a critical role in the postischemic inflammatory response after surgical decompression (12, 35). To determine if decompression surgery influences the extent of astrogliosis in an isoform-specific manner, we performed immunohistochemistry for the marker glial fibrillary acidic protein (GFAP) before decompression and 24 hours after decompression. We measured GFAP immunoreactivity over a constant area within the ventral horns of cervical spinal cord sections (Figure 10A). It was determined that before decompression, there were no isoform-specific differences between the E3-DCM and E4-DCM mice when normalized to their sham counterparts (*P* = 0.8173; mixed effects analysis with Sidak’s multiple comparisons; Figure 10B). However, 24 hours after decompression surgery, the E4-DCM mice had a significantly higher proportion of astrogliosis when compared with the E3-DCM mice (*P* = 0.0013; mixed effects analysis with Sidak’s multiple comparisons; Figure 10C).

## Discussion

This study investigates the potentially novel association between functional outcomes of DCM patients and the ApoE4 isoform, combining preclinical and clinical findings. We demonstrate that a significant proportion of DCM patients who are carriers of the ApoE4 isoform do not improve, and even deteriorate, after surgical decompression. This is a critical finding, as the current gold standard for DCM treatment is decompression surgery. To further investigate this association, we used a clinically relevant model of DCM (7) in genetically modified mice that possess the human ApoE gene and reproduced the progressive nature of cord compression seen in the human disease. In this model, we determined that in human ApoE4–knockin mice with DCM, the period after decompression surgery demonstrated a delayed neurological recovery and exacerbated peripheral and local inflammatory responses. These data revealed an isoform-specific role for microglia and astrocytes following decompression surgery. Our potentially novel data suggest a genetic mechanism that accounts for the significant heterogeneity in clinical and functional outcomes after surgical decompression in DCM patients. These findings open the door for novel personalized medicine strategies in these vulnerable, at-risk patients.

In this study, the mJOA scale was used to assess functionality in DCM patients pre- and postdecompression. The mJOA scale is the most widely accepted outcome measure for assessing DCM patients (36, 37). This scale assesses the severity of upper and lower limb motor dysfunction, sensory deficits, and sphincter dysfunction. Consistent with a previous study (21), we determined that ApoE4 carriers had a significantly lower level (Figure 1) of improvement compared with ApoE4 noncarriers, such that 28.6% of ApoE4 carriers deteriorated following decompression surgery compared with 0% of noncarriers. This is a stark difference to previous reporting that 9.3% of DCM patients exhibit functional decline postdecompression (7). Although the current study has a small sample size of patients, it highlights a critical need for further investigation into the pathological implications of the ApoE4 allele.

To complement our human clinical data examining the impact of ApoE4 on functional outcomes, we induced a DCM injury in human ApoE3– or ApoE4–knockin mice. This model of DCM simulates the progressive compression at C5-C6 spinal levels, which are the most commonly affected levels seen in the human disease, and allows for surgical decompression (32). We demonstrate that in ApoE4 mice surgical decompression was associated with increased inflammation and astrocyte reactivity as well as a delayed recovery in gait, forelimb grip strength, and hind limb mechanical sensitivity. Additionally, 24 hours postdecompression, ApoE4 mice exhibited differences in GM area, an exacerbated peripheral inflammatory response, and astrogliosis at an early decompressive time point. This was accompanied by increased activation of macrophage/microglial cells to the injury epicenter.

Several studies have shown that increased cytokine release by activated macrophages/microglia is harmful to the injured spinal cord and results in neuronal cell death and delayed recovery of motor functions (38–40). Moreover, the findings of the present study are consistent with previous studies suggesting an important role for ApoE4 in glia-mediated inflammation and subsequent neurological outcomes (23, 25, 41–45). Given the delayed neurological recovery in ApoE4 mice, these results indicate an isoform-specific immune response that may contribute to poor surgical outcomes in DCM through ApoE4- and glia-mediated mechanisms.

### ApoE and its role in glia-mediated inflammatory response.

Studies in AD and other neurological conditions have identified a crucial role for ApoE in glia-mediated inflammation and neurodegeneration. Data have shown that inflammation-induced microglia elicit the activation of neurotoxic reactive astrocytes, resulting in rapid neuronal death (45, 46). Shi and colleagues (45) demonstrated that a tauopathy mouse model expressing human ApoE4 produces a robust astrocytic activation, which leads to substantial neuronal loss. The findings of this study suggested neurons are more susceptible to degeneration in the presence of ApoE4, ultimately leading to further neuroinflammation. In these same mice, when assessing their microglial gene expression profiles, there was a significant upregulation of proinflammatory genes, such as *Clec7a*, *Tgm1*, *Olfr110*, and triggering receptor expressed on myeloid cells 2 (*Trem2*), in 9-month-old tauopathy mice expressing human ApoE4 (45).

Additionally, it has been suggested that ApoE and coexpressing gene networks, such as TREM2 and its signaling adapter DNAX-activating protein 12 (DAP12), significantly influence the regulation of glial cells and their ability to change molecular signature and functioning during injury and disease (25, 47, 48). Variants of TREM2 have been associated with a 2- to 4-fold increased risk of developing AD and are suggested to be crucial regulators of microglial activity (49–52). Krasemann and colleagues (25) identified a molecular signature for disease-associated microglia that is dependent on the endogenous murine ApoE (*mApoE*) binding to the TREM2-DAP12 receptor complex. This molecular signature was present in microglia derived from a mouse model of AD where its phenotypic switch from homeostatic to disease-associated microglia was accompanied by phagocytosis of apoptotic neurons and further progression of AD pathology.

Transcriptomic analysis of targeted-replacement mice expressing human ApoE4 has identified a significantly altered brain transcriptome in response to traumatic brain injury, specifically increasing the expression of genes related to immune response and phagocytosis, such as *Trem2*, *Dap12*, *Clec7a*, *Cd68*, and *Cx3cr1*. There were also astrocyte-specific genes, such as *Gfap*, *Aqp4*, and *Clu*, with increased expression in response to injury (30). Additionally, using weighted gene coexpression network analysis, Castranio and colleagues (30) revealed a network of genes highly associated with ApoE isoforms to be linked with the innate immune response. This network of genes demonstrated a shift toward increased expression in ApoE4 mice compared with ApoE3 mice. One of those genes included *Fyn* (*Fyn* proto-oncogene, Src family tyrosine kinase), a key regulator of proinflammatory processes via microglia-mediated neuroinflammation (53). The findings of this study support the notion that ApoE4, along with associated gene networks, produces an exacerbated proinflammatory cascade in response to injury.

### Current limitations.

Although the mJOA scale is the most widely accepted outcome measure, it has low sensitivity in detecting relevant functional change among patients. In the context of patients with mild DCM, this limitation may lead to problems in the scale’s ability to provide an accurate depiction of subtle changes in dysfunction that inform treatment strategy (54). However, the mJOA is still considered the most valid and responsive measure in assessing the recovery rate of DCM patients after surgical treatment (55). Additionally, this work will allow for broader identification of isoform-specific differences in functional outcomes after decompression surgery. For example, the influence of ApoE2, which has also been implicated in models of AD, may warrant investigation in DCM (41).

### Future directions.

We believe this is the first study that uses a preclinical rodent model to investigate the association between ApoE4 and DCM. Although the current study provides strong evidence for the important role ApoE4 plays in the regulation of the immune response and functional outcomes postdecompression, there remains an opportunity for further investigation into the specific mechanisms ApoE4 is affecting. For instance, it may be worth exploring TREM2-mediated pathways in the context of DCM through loss-/gain-of-function experiments. Studies in AD and other neurological diseases have examined the impact genetic ablation of TREM2 may have with respect to neuroinflammation and neurological decline. In mouse models of AD, it was shown that TREM2 deletion significantly decreases the number of plaque-associated microglia and inflammatory cytokines (56, 57). To further investigate the mechanistic pathways ApoE4 is affecting, it would be interesting to look at specific cell populations by utilizing human ApoE–knockin mice where ApoE4 is conditionally deleted in microglia (Tmem119-Cre) or astrocytes (GFAP-Cre) to determine the cellular source of ApoE4 influence.

Additionally, it is worth noting that activation of the immune response may not always be detrimental and thus should not be entirely suppressed. For instance, Yang and colleagues (58) demonstrated that a contused model of spinal cord injury (SCI) in *mApoE*-knockout mice led to an exaggerated inflammatory response with increased expression of nuclear factor κB (NF-κB) and cytokine levels such as IL-6 and IL-1β. Since the activation of NF-κB is known to promote the overproduction of inflammatory cytokines, the study examines whether inhibition of the NF-κB signaling pathway via pyrrolidine dithiocarbamate administration would reverse its harmful effects. This suggests *mApoE* deficiency increases the inflammatory response after SCI in a NF-κB–dependent manner. However, the same study claimed human ApoE4–knockin mice exhibited poor locomotor function after SCI compared with WT mice. Numerous studies also confirm ApoE4 plays a role in NF-κB–mediated transcription of genes associated with increased inflammatory cytokine release (59, 60), thus suggesting both endogenous *ApoE* and ApoE polymorphisms produce significant differences and a possible mechanistic pathway through which ApoE4 may influence DCM pathophysiology. Overall, this provides support for further mechanistic studies into the potential influence NF-κB–mediated pathways may have on ApoE4 in DCM.

### Conclusions.

The present study clearly depicts the important role ApoE4 plays in clinical outcomes and the immune response after surgical decompression for the treatment of DCM. These findings provide proof-of-concept data for further examination into a mechanistic influence of ApoE4 on DCM pathobiology. From a clinical perspective, this study suggests the possible benefit of development of a personalized medicine approach for an at-risk ApoE4^+^ patient population. This may entail screening of patients for the ApoE4 allele and require a more intensive antiinflammatory management strategy in the perioperative period.

## Methods

### Human subjects and study design

All study participants were recruited if they met the following inclusion criteria: (a) ages of 18–80 years old, (b) English speaking, (c) presenting with at least 1 clinical sign of spinal cord compression, (d) presenting with at least 1 neurological sign of spinal cord compression, (e) imaging confirmation of cervical spinal cord compression, and (f) no previous cervical spine surgery. Study participants were excluded if they had previous history (within the past 5 years) of cardiovascular disease, diabetes, poor liver function, poor kidney function, cancer, or stroke. Additionally, potential participants who were pregnant or had a recent history of substance abuse were excluded in order to limit potential confounding influences.

Once enrolled, demographic information and neurological assessment data were collected from all study participants at baseline and 12 months following surgery. For this analysis, the mJOA was used as the primary outcome measure. This investigator-administered, 18-point scale assesses upper and lower extremity motor function, sensory deficits, and sphincter dysfunction. Severity is scored by 3 levels, mild (score 15–18), moderate (score 12–14), and severe (score 0–11), with a minimum clinically important difference of 1 point for mild, 2 points for moderate, and 3 points for severe (61).

### Human blood collection and analysis

All blood was drawn at the blood clinic in Toronto Western Hospital. Blood samples were collected in a 10 mL EDTA-coated collection tube and immediately stored at –80°C until DNA extraction.

A 2-step cell lysis protocol was used to extract DNA from whole blood samples, as previously described (62). A phenol-chloroform method with ethanol precipitation for DNA isolation was used, as previously described (63). Briefly, a 1:1 ratio of DNA and phenol/chloroform/isoamyl alcohol (25:24:1) was prepared and carefully inverted. Samples were then centrifuged at room temperature for 5 minutes at 16,000*g*; the upper aqueous phase containing the purified DNA was transferred to a fresh tube. Reagents were added to the aqueous phase, glycogen (20 μg/μL), 7.5 M NH_4_OAc, and 100% ethanol, to precipitate the DNA from the sample overnight at –20°C. Samples were centrifuged at 4°C for 30 minutes at 16,000*g* to pellet the complementary DNA (cDNA). Supernatant was carefully removed, and 70% ethanol was added and centrifuged at 4°C for 2 minutes at 16,000*g*, which was repeated twice. The remaining cDNA pellet was dried at room temperature for 5–10 minutes and resuspended in TRIS buffer for storage. Finally, eluted DNA quantity and purity were determined using a NanoDrop 1000 spectrophotometer (Thermo Fisher Scientific).

### Genotyping

Utilizing real-time quantitative PCR methodology, the ApoE isoform of each patient was determined using TaqMan SNP genotyping assay (Applied Biosystems, Thermo Fisher Scientific), as per manufacturer’s instructions (64). Briefly, samples were aliquoted into 96-well plates and diluted in a mixture containing TaqMan Genotyping MasterMix (catalog 4371353) and 2 dye-labeled TaqMan probes, rs429358 (assay ID C_3084793_20) and rs7412 (assay ID C_904973_10) (Thermo Fisher Scientific). A pre-PCR and post-PCR plate read was performed using the Applied Biosystems 7900HT Fast Real-Time PCR System (Thermo Fisher Scientific) with standard thermal cycler conditions. Allelic discrimination analysis was carried out and processed using TaqMan Genotyper Software (Thermo Fisher Scientific).

### Animal experiments and study design

Mice were bred and housed at the Krembil Discovery Animal Research Facility at Toronto Western Hospital. Adult male and female breeding pairs for human ApoE4–knockin [B6(SJL)-Apoe^tm1.1(APOE*4)Adiuj^/J; stock 027894] and human ApoE3–knockin [B6.Cg-Apoe^em2(APOE*)Adiuj^/J; stock 029018] mice were purchased from The Jackson Laboratory (E4-KI and E3-KI, respectively). The E4-KI mice carry the humanized ApoE gene that encodes the isoform E4 sequence in place of the endogenous *Apoe* gene via targeted mutation methodology. The E3-KI mice are CRISPR/Cas9-generated mice that introduce a C to T nucleotide substitution into the humanized ApoE sequence of the Apoe^tm1.1(APOE*4)Adiuj^ allele to express the human ApoE3 isoform. All mice were genotyped to confirm homozygous expression of their respective human ApoE sequence.

Thirty-seven E4-KI and 35 E3-KI mice were used in this study, divided into 4 experimental groups: (group 1) E4-KI mice with DCM (E4-DCM; *n* = 21), (group 2) E4-KI mice with sham surgery (E4-Sham; *n* = 16), (group 3) E3-KI mice with DCM (E3-DCM; *n* = 20), and (group 4) E3-KI mice with sham surgery (E3-Sham; *n* = 15). Surgical induction of DCM was performed, between 8 and 10 weeks of age, and decompression was performed 6 weeks after induction (Figure 2). Mice were sacrificed at 24 hours, 72 hours, 3 weeks, and 5 weeks after surgical decompression. Both male and female mice were used and balanced between each experimental group. No sex-specific differences were found in the initial analysis; thus, sexes were grouped and analyzed together.

Sample sizes varied at different behavioral time points. The E3-DCM and E4-DCM experimental groups had an *n* = 17, the E3-Sham group had an *n* = 13, and the E4-Sham group had an *n* = 14 for all time points predecompression. Accounting for the multiple sacrifice time points, the sample sizes for each experimental group decreased. The E3-DCM, E4-DCM, E3-Sham, and E4-Sham groups had an *n* = 8 for 1–3 weeks postdecompression due to the 24-hour and 72-hour sacrifice time points. Then finally, the E3-DCM, E4-DCM, and E3-Sham groups had an *n* = 4, and the E4-Sham group had an *n* = 3, for 4–5 weeks postdecompression to account for the 3-week sacrifice time point. A total of 3 animals were lost due to unfortunate conditions or circumstances, such as malocclusion and sudden death unrelated to the DCM injury or decompression surgery. One animal was from the E3-DCM experimental group for the 72-hour time point, another was from the E3-Sham group at the 72-hour time point, and 1 animal was from the E4-Sham group for the 24-hour time point. All experimental procedures were performed by one investigator who was blinded to the experimental groups.

### DCM induction and decompression surgery

The DCM mouse model was adapted from a previously established rat DCM model (32). Briefly, a midline incision from C4 to T1 was made to expose and dissect the superficial muscle layers where a retractor was set in place to expose the spinous processes. From C4 to C7, the ligaments surrounding each spinal lamina were carefully removed using sharp forceps. Focusing on C5 and C6 laminae, the inferior surface was scratched using a microhook to activate bone remodeling. A sheet of polyaromatic ether was folded and placed under the C5-C6 laminae to act as a scaffold for the bone mineral deposition and subsequent progressive compression of the spinal cord. For sham animals, all procedures were kept consistent, with the exception of the polyaromatic ether material implantation. To simulate and control for any potential damage associated with implantation, the material was inserted under the C5-C6 laminae for 30 seconds and then removed.

During surgical decompression, as previously described (12, 32), the C5-C6 laminae were removed using microscissors to expose the spinal cord underneath and relieve its compression. Dural sac pulsation was observed to confirm a successful decompression with no cerebrospinal fluid leakage. Sham animals underwent the same surgical procedure, with the exception of decompression. Muscle and skin layers were sutured. A subcutaneous buprenorphine injection (0.1 mg/kg) was given for postoperative pain, and saline injection (0.1 mg/kg) was given for dehydration. Buprenorphine injections were continued twice a day for 3 days. All animals were examined 24 hours after surgery for any signs of neurological deficits that may be caused by damage to the spinal cord or roots during the procedure. Any indication of a deficit resulted in the exclusion of the animal from the study.

### Neurobehavioral assessments

All behavioral tests were conducted weekly beginning 1 week after surgical induction of DCM and after decompression surgery. Data collection was performed at the same time of day by the same experimenter.

#### Analysis of spatiotemporal gait.

Using the CatWalk XT walkway system (Noldus), gait deficits were analyzed in all mice pre- and postdecompression. Mice were permitted to walk from one end of the device to the other, while a camera below recorded their locomotion in real time. We focused the analysis on the following parameters: stride length, cadence, and step regularity index. Only uninterrupted runs with a minimum of 3 continuous step cycles were considered for analysis. For each animal, a total of 5 compliant runs were collected.

#### Analysis of grip strength.

The forelimb wire hang test was used to evaluate neuromuscular grip strength pre- and postdecompression in all mice. Briefly, animals were placed on a standard wire cage top that was held 15 cm above a pillowed cage. Once the animals gripped the lid, it was slowly inverted; the latency for each animal to drop was recorded.

#### Analysis of hind paw mechanical sensitivity.

Using an electronic von Frey apparatus, mechanical sensitivity in hind paws was assessed, as previously described (33). The testing apparatus was a nonhygroscopic polypropylene von Frey tip (0.8 mm diameter) attached to an electronic probe with readout display to record the force/weight at which the mouse retracted from the tip. To begin data collection, the tip of the probe stimulated the middle of the left and right hind paws (2 times each for a total of 4 probes). Mice were given ample time between each trial to decrease their level of stress and irritation of the target site.

### Tissue collection and processing

At experimental endpoints, mice were transcardially perfused with 50 mL of ice-cold 1× PBS and subsequently with 50 mL of fresh 4% paraformaldehyde. Once perfused, spinal cords were dissected using microscissors and placed in a postfix solution made of 4% paraformaldehyde with 10% sucrose for 5 hours. Spinal cords were subsequently washed with 1× PBS and placed in a cryoprotection solution of 30% sucrose in 1× PBS overnight. Subsequently, spinal cords were cryosectioned using a Leica cryostat at a temperature of approximately –20°C. Sections were cut cross-sectionally at 30 μm thickness, placed on Fisherbrand Superfrost Plus microscope slides (Thermo Fisher Scientific), and stored at –80°C prior to immunohistochemistry.

### Serum collection and processing

Prior to perfusion, all animals received a cardiac puncture to collect whole blood samples. Samples were allowed to clot at room temperature for 30 minutes. Subsequently, they were centrifuged at 3000 rpm for 10 minutes, and the serum was extracted and stored in –80°C until processing.

Levels of cytokines of interest were assessed for protein concentration using mouse 31-panel high-throughput profiling discovery system with Luminex xMAP technology (Eve Technologies). Frozen serum samples were thawed and aliquoted (75 μL per sample) to microcentrifuge tubes to be diluted (1:1) with 1× PBS (pH 7.4). Samples were subsequently packaged and shipped to Eve Technologies, from which results were collected and provided.

### Histopathology

Coronal sections (30 μm thickness) were stained with the cellular stain HE and myelin-selective pigment LFB. Images were taken at original magnification 10× on the Leica DMRB epifluorescence microscope. GM and WM were clearly contrasted by the stain and were manually demarcated and quantified in Fiji. WM/GM ratio was calculated by taking the quotient of the total WM area over the GM area for each individual section.

### Immunohistochemistry

Coronal sections (30 μm thickness) were blocked with 5% (*w/v*) skim milk powder, 1% bovine serum albumin, and 0.3% Triton X-100 (*v/v*) in 1× PBS for 1 hour at room temperature in a humidified chamber. Subsequently, the blocking solution was aspirated off the slides, and primary antibodies, GFAP (C9205, Cy3-conjugated, 1:1000), Iba-1 (PA5-21274; 1:500), and CD68 (4-0681-80; 1:300; all from Thermo Fisher Scientific), diluted with fresh blocking solution, were applied onto the tissue sections. Primary antibodies were incubated in a light-shielded humidified chamber and placed in a 4°C fridge overnight. Slides were washed with 1× PBS 3 times for 10 minutes each. Secondary antibodies (A-21244 goat anti-rabbit IgG Alexa Fluor 647 and A-11006 goat anti-rat IgG Alexa Fluor 488; 1:1000; both from Invitrogen, Thermo Fisher Scientific) and DAPI nuclear counterstain (1:500, Invitrogen, Thermo Fisher Scientific) were diluted in fresh blocking solution and applied to the tissue sections to be incubated at room temperature for 1–2 hours. Again, slides were washed with 1× PBS 3 times for 10 minutes each and subsequently coverslipped using Mowiol aqueous mounting medium (MilliporeSigma).

### Microscopy

Eight cryosections from each experimental animal at an interval of 240 μm around the compression epicenter were imaged at original magnification 20× on a Zeiss Axioplan2 epifluorescence microscope using AxioVision 4.6 software. The dorsal and ventral horns from 1 consistent side were captured in 2 images for each animal to quantify DAPI^+^, CD68^+^, and Iba-1^+^ cells. Averaged Iba-1^+^, CD68^+^, and Iba-1^–^CD68^+/+^ colocalization cell counts were normalized to the average DAPI^+^ cell counts. For GFAP^+^ area fraction, 1 consistent area at the gray-white matter intersection was captured for each animal. This image was processed in Fiji to remove non-GFAP^+^ background by size (Subtract Background, rolling = 10) and thresholded (Threshold, grayscale cutoff = 11). The resultant images were quantified, and the GFAP^+^ area fraction was reported.

### Cell counting and colocalization

Eight-bit images were passed through a median filter (radius = 2), and DAPI^+^, CD68^+^, and Iba-1^+^ cells were identified and counted using the find maxima function (prominence = 10) within Fiji. For the count of Iba-1^+^CD68^+^ colocalization, background subtraction was performed (rolling = 20) on the 8-bit images of Iba-1 and CD68. Using the image calculator, pixels that existed on both the images were summed. These images were then passed through a median filter (radius = 2) and the find maxima function (prominence = 60) to determine colocalized cell counts.

### Statistics

All data were analyzed and graphed using GraphPad Prism (version 8). Human clinical data were analyzed using a Mann-Whitney nonparametric, 2-tailed *t* test between 2 groups, ApoE4 noncarriers and ApoE4 carriers, postoperatively. Demographic and clinical characteristics of ApoE4 noncarriers and ApoE4 carriers were described using frequencies, counts, and χ^2^ tests for categorical variables and Mann-Whitney *U* tests for continuous variables.

All animal behavior, including CatWalk, forelimb wire hang test, and hind limb von Frey filament test, were analyzed using a mixed effects model allowing for repeated measures and multiple comparisons with varying sample sizes between groups. GraphPad Prism 8 offers an alternative to computing repeated measures ANOVA with a data set that has missing values, in this case because of multiple sacrifice time points. This alternative solution analyzes repeated measures data by fitting a mixed effects model comparable to a repeated measures ANOVA. Additionally, an assessment of normality was performed using the Shapiro-Wilk test for each group in order to assume all data fit a Gaussian distribution. All data were separated into predecompression and postdecompression graphs depicting all behavioral time points and comparing all experimental groups. The histopathology data were analyzed using a 2-way ANOVA with Sidak’s post hoc correction for multiple comparisons. Cytokine levels were analyzed via 1-way ANOVA with Sidak’s post hoc correction for multiple comparisons. Levene’s test for homogeneity of variance was performed to assume equal variance among the data. Time points were analyzed separately comparing all experimental groups at 1 specific time point, before decompression or 24 hours postdecompression. Immunohistochemistry data for Iba-1^+^ and CD68^+^ cell counts were analyzed using a 1-way ANOVA with Sidak’s post hoc correction for multiple comparisons. Normality assessments of the 24-hour postdecompression Iba-1^+^CD68^+^ colocalization data revealed that the E4-DCM group did not have a normal distribution. We then performed the Grubbs’s outlier test (α = 0.05) to see if outliers existed in the data set. From this, the test removed 2 samples total, 1 from the E3-DCM group and 1 from the E4 DCM group (total *n* = 3/group). After outlier removal, the 24-hour postdecompression Iba-1^+^CD68^+^ colocalization data passed the Shapiro-Wilk normality assessments. Last, immunohistochemistry data for GFAP were analyzed using a mixed effects model with Sidak’s post hoc correction for repeated measures. The DCM data were normalized to their respective shams. Results were considered significant if they had a *P* value less than 0.05.

### Study approval

#### Human experiments.

All study protocols were reviewed and approved by the University Health Network Research Ethics Board, Toronto, Ontario, Canada. Written informed consent from study participants was obtained during initial enrollment at Toronto Western Hospital, Ontario, Canada.

#### Animal experiments.

All animal procedures and experiments were designed and conducted in accordance to the Canadian Council for Animal Care guidelines and approved by the University Health Network Animal Care Committee, Toronto, Ontario, Canada.

## Author contributions

AD was responsible for conceptualization, methodology, conducting experiments, data curation, formal analysis, investigation, and writing — original draft; JH was responsible for investigation, conducting experiments, data curation, and writing — reviewing and editing; STB was responsible for conducting experiments and data curation; WY was responsible for conducting experiments; AML was responsible for conceptualization, investigation, and data curation; and MGF was responsible for conceptualization, resources, writing — reviewing and editing, supervision, and funding acquisition.

## Supplementary Material

Supplemental data

## Figures and Tables

**Table 1:**
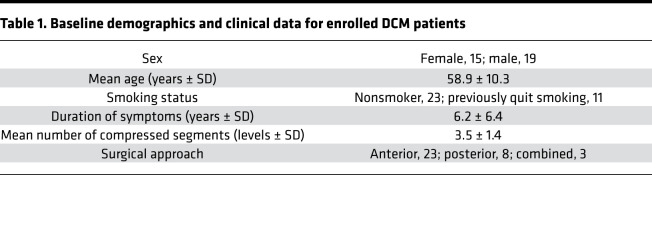
Baseline demographics and clinical data for enrolled DCM patients

**Table 2:**
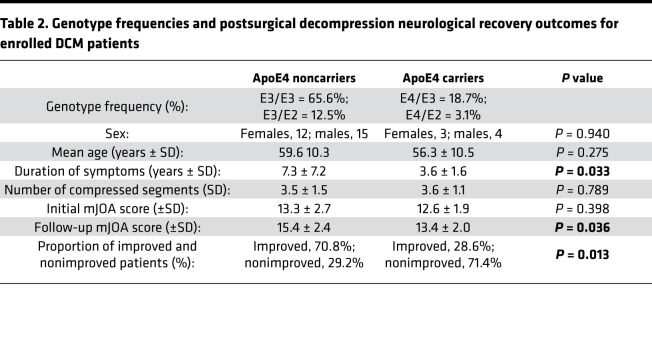
Genotype frequencies and postsurgical decompression neurological recovery outcomes for enrolled DCM patients

**Table 3:**
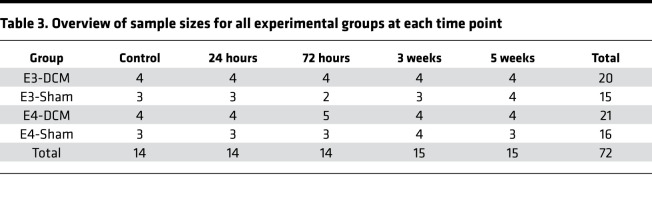
Overview of sample sizes for all experimental groups at each time point

## Version 1 08/09/2021

Electronic publication
