# Supplementary material for: The influence of ApoE4 on the clinical outcomes and pathophysiology of degenerative cervical myelopathy
Source: JCI Insight. 2021 Aug 9;6(15):e149227. doi: 10.1172/jci.insight.149227 (PMC8410082; doi:10.1172/jci.insight.149227)
Supplement: Supplemental data [file jciinsight-6-149227-s231.pdf]

Supplemental File

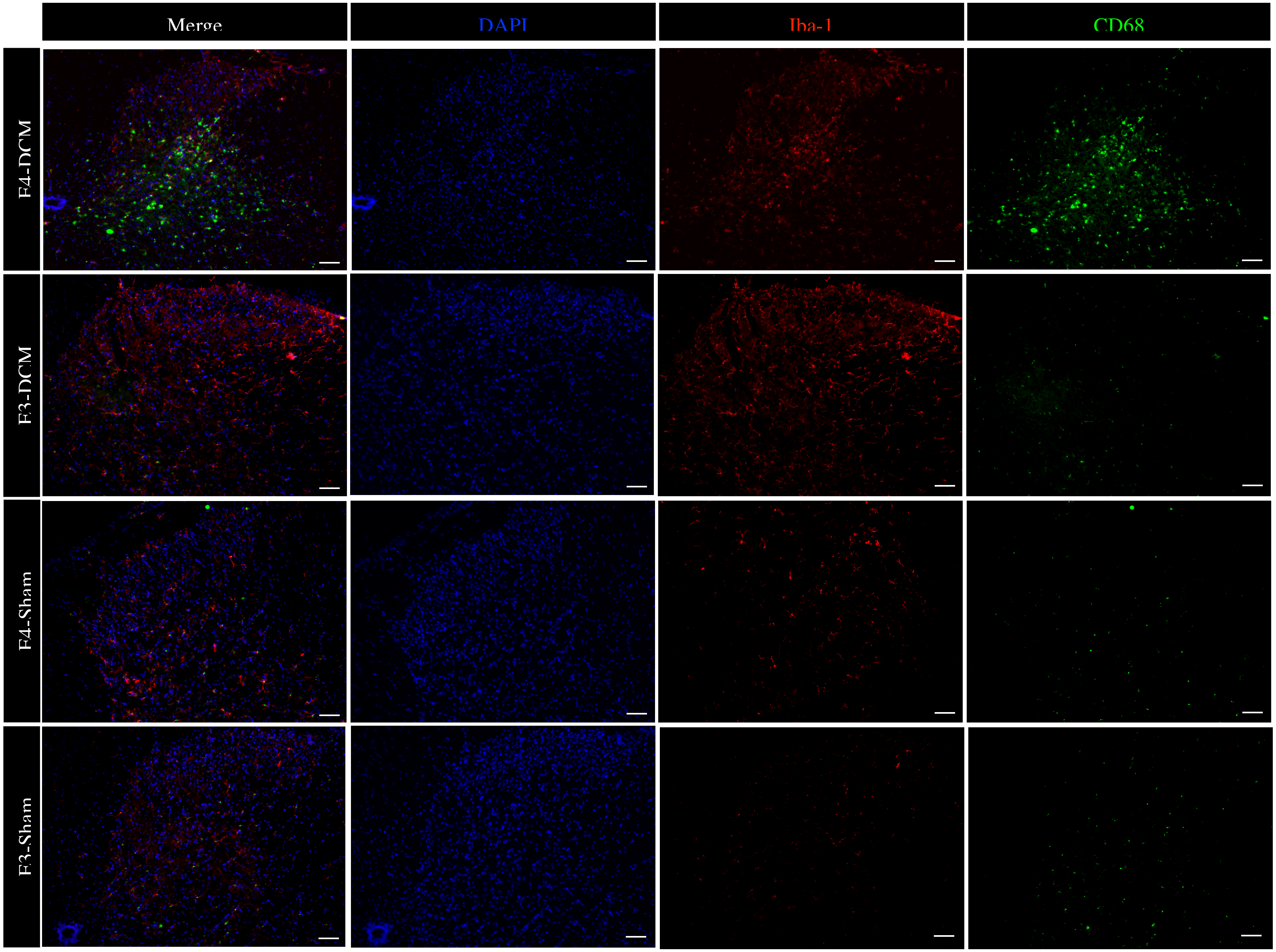

Supplementary Figure 1. Representative images from immunohistochemistry experiment used for cell quantification. Images are depicting Iba-1^{+} (red) and CD68^{+} (green) cells with nuclear countersain DAPI (blue) present in the dorsal horn regions spinal cord sections around the injury epicenre. Scale bars: 100μm.

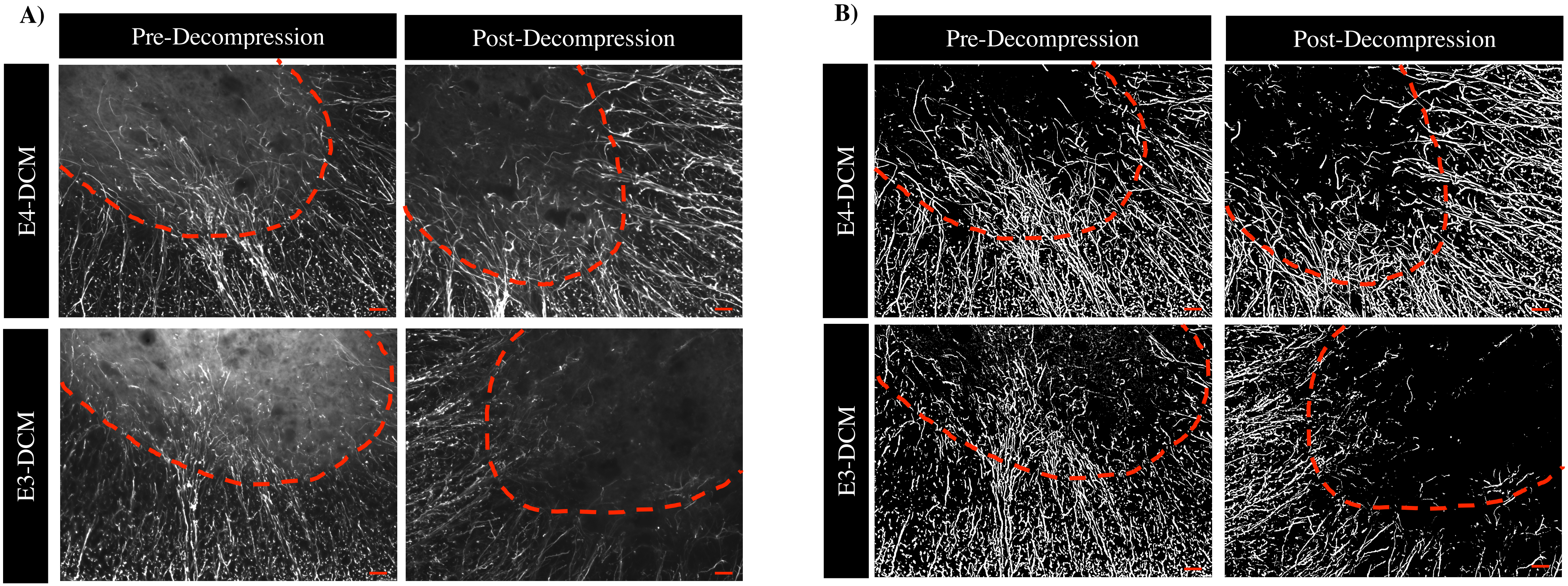

Supplementary Figure 2. Representative images from immunohistochemistry experiment used for GFAP quantification. One consistent area at the gray-white matter intersection were captured for each animal. (A) Original images of ventral horns from E3-DCM and E4-DCM mice before decompression and 24-hours after decompression. (B) Representative images used for GFAP quantification. Images were processed in Fiji to remove non-GFAP+ background by size (Subtract Background, rolling = 10) and thresholded (Threshold, greyscale cut-off = 11). The resultant images were quantified, and the $GFAP^+$ area fraction was reported. The red dotted lines delineate the ventral horns. Scale bars: 25µm.
